# Fuzzy fractional-order model of the novel coronavirus

**DOI:** 10.1186/s13662-020-02934-0

**Published:** 2020-09-05

**Authors:** S. Ahmad, A. Ullah, K. Shah, S. Salahshour, A. Ahmadian, T. Ciano

**Affiliations:** 1 Department of Mathematics, University of Malakand, Chakdara Dir (Lower), Khyber Pakhtunkhawa Pakistan; 2 Faculty of Engineering and Natural Sciences, Bahcesehir University, Istanbul, Turkey; 3 Institute of IR 4.0, The National University of Malaysia, 43600 Bangi, Selangor Malaysia; 4 Department of Law, Economics and Human Sciences & Decisions Lab, University Mediterranea of Reggio Calabria, Reggio Calabria, Italy

**Keywords:** Approximate solutions, Fuzzy number, Fuzzy fractional order derivative, Coronavirus infection system, Adomian decomposition method

## Abstract

In this paper, a novel coronavirus infection system with a fuzzy fractional differential equation defined in Caputo’s sense is developed. By using the fuzzy Laplace method coupled with Adomian decomposition transform, numerical results are obtained for better understanding of the dynamical structures of the physical behavior of COVID-19. Such behavior on the general properties of RNA in COVID-19 is also investigated for the governing model. The results demonstrate the efficiency of the proposed approach to address the uncertainty condition in the pandemic situation.

## Introduction

Recently, the whole globe has been suffering from a novel coronavirus pandemic, which was named “2019 novel coronavirus”, abbreviated by “2019-nCoV”, and claimed to outbreak for the first time in Wuhan city, central China [1]. It has been observed that 2019-nCoV is transmitted from animal to human; as many infected claimed that they had been infected due to a local fish and wild animal market in Wuhan as early as 28 November [2]. Soon after, some researchers confirmed that the transmission also happens from a person to a person [3]. According to the data reported by WHO (World Health Organization), on March 21, 2020, the reported laboratory confirmed human infections in 187 countries, territories, or areas around the world have reached more than 292,142, including 12,784 death cases [4]. Even in some countries, like Italy and Spain, the death rate was as high as almost 0.066. This verifies the severity and high infectivity of 2019-nCoV. It is confirmed that most people infected with 2019-nCoV will experience mild to moderate respiratory illness, such as breath difficulty, low fever, sick, cough, and other symptoms. However, other symptoms such as gastroenteritis and neurological diseases of varying severity have also been reported [5]. The 2019-nCoV is transmited mainly through droplets from the nose when an infected person coughs or sneezes. Once a person breaths the droplets from infected people in the air, he/she will be exposed to the danger of getting the infection. As a result, the best way to prevent the virus is to avoid meetings and touching other people. For this purpose, the Chinese government decided to lock down Wuhan city and cut or limit the transportation system of the country, including airplanes, trains, buses, and private cars, etc., to control population flow and movement. People were required to stay at home and get body temperature taken each day. Respirators were advocated to be worn if people had to go out. With the transmission and outbreak of 2019-nCoV around the world, more governments joined the antivirus battle by following the Chinese government. It was heard that more and more countries started to release regulations to ban international travel, close schools, shopping malls, and companies. The 2019-nCoV pandemic has lead to a serious economic damage in the whole world, and it has also been a great ordeal for the administrations of countries and even to all human beings. A great number of doctors and researchers also devoted themselves to the antipandemic war and did researches based on their expertise. They looked into 2019-nCoV from various points of view, such as virology, infectious diseases, microbiology, public environmental occupational health, veterinary sciences, sociology, media studies, political economics, etc. China, USA, and Korea are the leading countries on the 2019-nCoV research because the early outbreak of virus urged them to start relevant research immediately. A group of researchers studied the origin of 2019-nCoV. Initially, it was said that bats are the origin of 2019-nCoV, which is similar to SARS (Severe Acute Respiratory Syndrome), an epidemic which broke out in China and other regions of the world in 2003 [6, 7]. Then some researchers compared 2019-nCoV with SARS and MERS (Middle East Respiratory Syndrome) from 2012 to prove the possibilities to learn lessons from the two pandemics happened before in the human history. According to Lu, SARS-CoV, MERS-CoV, and 2019-nCoV all belongs to the same family of Betacoronavirus genus [8]. According to Zhou, previous research indicates that 2019-nCoV has high similarity to SARS-CoV, which is supported by the full-length genome phylogeny analysis, and therefore, has the putative similar cell entry mechanism and human cell receptor usage [9]. Xiaolong and Mose also considered the high identity of RBD (Receptor Binding Domain) in 2019-nCoV and SARS-CoV, and raised the idea that the SARS-CoV specific human antibody, CR3022, could bind potently with 2019-nCoV RBD, KD of 6.3 nM, which indicates that the difference within the RBD of SARS-CoV and 2019-nCoV incorporates a crucial influence on the cross-reactivity of neutralizing antibodies, which is still necessary to develop novel monoclonal antibodies that would bind specifically to 2019-nCoV RBD [10]. Based on the previous studies on SARS-Cov immunological system and structures, Syed et al. determined SARS-CoV-derived B lymphocyte epitopes and T cell epitopes experimentally, and located that they are similar and comprise no mutation within the available 2019-nCoV sequences, which is critical to narrow down the hunt for potent targets for an efficient vaccine against the 2019-nCoV. Some researchers put their focus on the transmission of 2019-nCoV virus among humans and its identification. It’s well accepted that human-to-human transmission is leading to the rapid growth of infections. Ahmed claimed that viral strains from the infected people of the area have been sequenced; but only little genetic variation was found, implying that they have descended from a common ancestor [11]. On the other hand, Zhou argued that sequences of the seven conserved viral replicase domains in ORF 1ab show 94.6% similarity in 2019-nCoV and SARS-CoV [9]. Chaudhury et al. proved that computational protein–protein docking with accurate, physics-based energy functions is able to reveal the native-like, low-energy protein–protein complex from the unbound structures of two individual, interacting protein components [12]. In our work we try to investigate 2019-nCoV infection system mathematically. The fuzzy Laplace transform based on Adomian decomposition is employed to obtain the numerical results which can be helpful for the understanding of the dynamical structures of the physical behavior of 2019nCoV. We define the system of six equations illustrating the outbreak of the coronavirus in the form of nonlinear fractional order differential equations (FODEs), involving the susceptible people $S_{k}(t)$, the exposed population $E_{k}(t)$, total infected strength $I_{k}(t)$, asymptotically infected population $A_{k}(t)$, the total number of humans recovered $R_{k}(t)$, reservoir $M_{k}(t)$, and corresponding interaction, which are presented as follows [13]:

1 $$ \textstyle\begin{cases} D_{t}^{\gamma }S_{k}(t) =n_{k}-m_{k}S_{k}-b_{k}S_{k}(I_{k}+\kappa A_{k})-b_{k}S_{k}M_{k}, \\ D_{t}^{\gamma }E_{k}(t) =b_{k}S_{k}(I_{k}+\delta A_{k})+b_{l}S_{k}M-(1- \delta _{k})\omega _{k}E_{k}-\delta _{k}\omega _{k}^{\prime }E_{k}-m_{k}E_{k}, \\ D_{t}^{\gamma }I_{k}(t) =(1-\delta _{k})\omega _{k}E_{k}-(\gamma _{k}+m_{k})I_{k}, \\ D_{t}^{\gamma }A_{k}(t) =\delta _{k}\omega _{k}^{\prime }E_{k}-(\gamma _{k}^{\prime }+m_{k})A_{k}, \\ D_{t}^{\gamma }R_{k}(t) =\gamma _{k}I_{k}+\gamma _{k}^{\prime }A_{k}-m_{k}R_{k}, \\ D_{t}^{\gamma }M_{k}(t) =\xi I_{k}+\eta A_{k}-\nu M_{k}, \end{cases} $$

where $n_{k}$ represents the rate of birth, $m_{k}$ represents the death of infected population, $b_{k}$ represents the transmission coefficient, $b_{l}$ represents disease transmission coefficient, *κ* is transmissibility multiple, $\omega _{k}$ and $\omega _{k}^{\prime }$ denote signified incubation period, $\gamma _{k}$ and $\gamma _{k}^{\prime }$ represent the recovery rate of $I_{k}$ and $A_{k}$, respectively, *ξ* and *η* denote the influence of the virus from $I_{k}$ and $A_{k}$ to $M_{k}$, and *ν* represents the rate of eliminating the virus from $M_{k}$. The parameters are explained in Table 1.

In the last few years, modern calculus and DEs have been extended to fuzzy calculus and FODEs [14–18], respectively. Then FODEs were extended to fuzzy FODEs [19–21]. FODEs and fuzzy integral equations have been studied by many researchers to establish the existence and uniqueness theory of solutions [22–27]. When dealing with fuzzy FODEs, it is really tedious to compute more precise solutions to every fuzzy FODE. A lot of efforts have been made by mathematicians in solving fuzzy FODEs by using various methods like perturbation method, integral transform methods, as well as spectral techniques [28–33]. Some researchers performed stability analysis of fuzzy DEs [34]. Here, we are going to investigate model (1) with a fuzzy fractional-order derivative where the uncertainty lies in the initial data. For $0<\gamma \leq 1$,

2 $$ \textstyle\begin{cases} D_{t}^{\gamma }\mathcal{Y}_{k}(t) =\tilde{n}_{k}-\tilde{m}_{k} \mathcal{Y}_{k}-\tilde{b}_{k}\mathcal{Y}_{k}(\mathcal{I}_{k}+ \tilde{\kappa }\mathcal{A}_{k})-\tilde{b}_{k}\mathcal{Y}_{k} \mathcal{M}_{k}, \\ D_{t}^{\gamma }\mathcal{V}_{k}(t) =\tilde{b}_{k}\mathcal{Y}_{k}(I_{k}+ \tilde{\delta }\mathcal{A}_{k})+\tilde{b}_{l}\mathcal{Y}_{k} \mathcal{M}-(1-\tilde{\delta }_{k})\tilde{\omega }_{k}\mathcal{V}_{k}- \tilde{\delta }_{k}\tilde{\omega }_{k}^{\prime }\mathcal{V}_{k}-\tilde{m}_{k} \mathcal{V}_{k}, \\ D_{t}^{\gamma }\mathcal{I}_{k}(t) =(1-\tilde{\delta }_{k}) \tilde{\omega }_{k}\mathcal{V}_{k}-(\tilde{\gamma }_{k}+\tilde{m}_{k}) \mathcal{I}_{k}, \\ D_{t}^{\gamma }\mathcal{A}_{k}(t) =\tilde{\delta }_{k}\tilde{\omega }_{k}^{\prime } \mathcal{V}_{k}-(\tilde{\gamma }_{k}^{\prime }+\tilde{m}_{k})\mathcal{A}_{k}, \\ D_{t}^{\gamma }\mathcal{R}_{k}(t) =\tilde{\gamma }_{k}\mathcal{I}_{k}+ \tilde{\gamma }_{k}^{\prime }\mathcal{A}_{k}-\tilde{m}_{k}\mathcal{R}_{k}, \\ D_{t}^{\gamma }\mathcal{M}_{k}(t) =\tilde{\xi }\mathcal{I}_{k}+ \tilde{\eta }\mathcal{A}_{k}-\tilde{\nu }\mathcal{M}_{k}, \end{cases} $$

associated to fuzzy initial condition, for $\alpha \in [0,1]$,

$$\begin{aligned}& \tilde{\mathcal{Y}}(0,\alpha ) = \bigl(\underline{\mathcal{Y}}(0, \alpha ), \overline{\mathcal{Y}}(0,\alpha ) \bigr), \\& \tilde{\mathcal{V}}(0,\alpha ) = \bigl(\underline{\mathcal{V}}(0, \alpha ), \overline{\mathcal{V}}(0,\alpha ) \bigr), \\& \tilde{\mathcal{I}}(0,\alpha ) = \bigl(\underline{\mathcal{I}}(0, \alpha ), \overline{\mathcal{I}}(0,\alpha ) \bigr), \\& \tilde{\mathcal{A}}(0,\alpha ) = \bigl(\underline{\mathcal{A}}(0, \alpha ), \overline{\mathcal{A}}(0,\alpha ) \bigr), \\& \tilde{\mathcal{R}}(0,\alpha ) = \bigl(\underline{\mathcal{A}}(0, \alpha ), \overline{\mathcal{A}}(0,\alpha ) \bigr), \\& \tilde{\mathcal{M}}(0,\alpha ) = \bigl(\underline{\mathcal{A}}(0, \alpha ), \overline{\mathcal{A}}(0,\alpha ) \bigr). \end{aligned}$$

Regarding the above explanations and to address the current uncertain situation, we were motivated to propose a novel coronavirus infection system under fuzzy fractional calculus. In fact, considering the proposed model which also enhances the physical behavior of such an infection system, we ensure that the model is closer to the real behavior of a system evolving the general properties of RNA in COVID-19.

## Preliminaries

### Definition 1

([35, 36])

Let $\mu :\mathbb{R}\rightarrow [0,1]$ be a fuzzy set of the real line satisfying the following properties:

(i) *μ* is normal (for any $a_{0}\in \mathbb{R}; \mu (a_{0})=1$);
(ii) *μ* is upper semicontinuous on $\mathbb{R}$ ($\forall \varepsilon >0\ \exists \delta >0 \ni \vert \mu (a)-\mu (a_{0}) \vert <\varepsilon , \vert a-a_{0} \vert <\delta $);
(iii) *μ* is convex ($\mu (\kappa a+(1-\kappa )b)\geq (\mu (a)\wedge \mu (b))\ \forall \kappa \in [0,1], a,b\in \mathbb{R} $);
(iv) $\operatorname{cl}\{a\in \mathbb{R}, \mu (a)>0\}$ is compact.

Then it is called a fuzzy number.

### Definition 2

([35])

On a fuzzy number *μ*, the *p*-level set is defined by

$$ [\mu ]^{p}=\bigl\{ x\in \mathbb{R} : \mu (x)\geq p\bigr\} , $$

where $p\in (0,1]$ and $x\in \mathbb{R}$.

### Definition 3

([35, 36])

Let $[\underline{\mu }(\vartheta ), \overline{\mu }(\vartheta )]$ be the parametric form of a fuzzy number *μ*, where $0\leq \vartheta \leq 1$, which satisfies the following properties:

(i) $\underline{\mu }(\vartheta )$ is left continuous, bounded, and increasing function over $(0,1]$, and right continuous at 0.
(ii) $\overline{\mu }(\vartheta )$ is right continuous, bounded, and decreasing over $[0,1]$, and right continuous at 0.
(iii) $\underline{\mu }(\vartheta )\leq \overline{\mu }(\vartheta )$.

Also, if $\underline{\mu }(\vartheta )=\overline{\mu }(\vartheta )=0$, then *ϑ* is called a crisp number.

### Definition 4

([31])

Consider a mapping $\rho :E\times E\rightarrow \mathbb{R}$ and let $v=(\underline{v}(\vartheta ),\overline{v}(\vartheta ))$ and $w=(\underline{w}(\vartheta ),\overline{w}(\vartheta ))$ be two fuzzy numbers in their parametric form. The Hausdorff distance between *v* and *w* is defined by

$$ \rho (v,w)=\sup_{\vartheta \in [0,1]} \bigl[ \text{max} \bigl\{ \bigl\vert \underline{v}(\vartheta )-\underline{w}( \vartheta ) \bigr\vert , \bigl\vert \overline{v}(\vartheta )-\overline{w}( \vartheta ) \bigr\vert \bigr\} \bigr]. $$

In *E*, the metric *ρ* has the following properties:

(i) $\rho (v+\upsilon ,w+\upsilon )=\rho (v,w)$ for all $v,\upsilon ,w\in E$;
(ii) $\rho (v\varrho ,w\varrho )= \vert \varrho \vert \rho (v,w)$ for all $v,w\in E$, $\varrho \in \mathbb{R}$;
(iii) $\rho (v+\xi ,w+\varsigma )\leq \rho (v,w)+\rho (\xi ,\varsigma )$ for all $v,w,\xi ,\varsigma \in E$;
(iv) $(E,\rho )$ is a complete metric space.

### Definition 5

([31])

Let $\tau _{1},\tau _{2}\in E$. If there exist $\tau _{3}\in E$ such that $\tau _{1}=\tau _{2}+\tau _{3}$ then $\tau _{3}$ is said to be the *H*-difference of $\tau _{1}$ and $\tau _{2}$, denoted by $\tau _{1}\ominus \tau _{2}$.

### Definition 6

([31])

Let $\varTheta :\mathbb{R}\rightarrow E$ be a fuzzy mapping. Then *Θ* is called continuous if for any $\epsilon >0\ \exists \delta >0$ and a fixed value of $\lambda _{0}\in [\zeta _{1},\zeta _{2}]$, we have

$$ \rho \bigl(\varTheta (\lambda ),\varTheta (\lambda _{0})\bigr)< \epsilon \quad \text{whenever } | \lambda -\lambda _{0}| < \delta . $$

### Definition 7

([28, 31])

Let *Φ* be a continuous fuzzy function on $[0,b]\subseteq \mathbb{R}$, a fuzzy fractional integral in Riemann–Liouville sense corresponding to *t* is defined by

$$ I^{\kappa }\varPhi (t)=\frac{1}{\varGamma (\kappa )} \int _{0}^{t}(t- \zeta )^{\kappa -1}\varPhi (\zeta )\,d\zeta,\quad \text{where } \kappa , \zeta \in (0,\infty ). $$

Further, if $\varPhi \in C^{F}[0,b]\cap L^{F}[0,b]$, where $C^{F}[0,b]$ and $L^{F}[0,b]$ are the spaces of fuzzy continuous functions and fuzzy Lebesgue integrable functions, respectively, then fuzzy fractional integral is defined as

$$ \bigl[I^{\kappa }\varPhi (t)\bigr]_{p}= \bigl[I^{\kappa } \underline{\varPhi }_{P}(t),I^{ \kappa }\overline{\varPhi }_{p}(t) \bigr],\quad 0\leq p\leq 1, $$

where

$$\begin{aligned}& I^{\kappa }\underline{\varPhi }_{p}(t) = \frac{1}{\varGamma (\kappa )} \int _{0}^{t}(t-\zeta )^{\kappa -1} \underline{\varPhi }_{p}(t)\,d\zeta ,\quad \kappa ,\zeta \in (0,\infty ), \\& I^{\kappa }\overline{\varPhi }_{p}(t) =\frac{1}{\varGamma (\kappa )} \int _{0}^{t}(t-\zeta )^{\kappa -1} \overline{\varPhi }_{p}(t)\,d\zeta ,\quad \kappa ,\zeta \in (0,\infty ). \end{aligned}$$

### Definition 8

([31])

If a fuzzy function $\varPhi \in C^{F}[0,b]\cap L^{F}[0,b]$ is such that $\varPhi =[\underline{\varPhi }_{p}(t), \overline{\varPhi }_{p}(t)]$, $0 \leq p\leq 1$ and $t_{1}\in (0,b)$, then the fuzzy fractional Caputo’s derivative is defined as

$$ \bigl[D^{\beta }\varPhi (t_{0}) \bigr]{}_{p}= \bigl[D^{\beta } \underline{\varPhi }_{p}(t_{0}),D^{\beta } \overline{\varPhi }_{p}(t_{0}) \bigr],\quad 0\leq \beta \leq 1, $$

where

$$\begin{aligned}& D^{\beta }\underline{\varPhi }_{p}(t_{0}) = \frac{1}{\varGamma (n-\beta )} \biggl[ \int _{0}^{t}(t-\zeta )^{n- \beta -1} \frac{d^{n}}{d\zeta ^{n}}\underline{\varPhi }_{p}(\zeta ) \,d\zeta \biggr]_{t=t_{0}}, \\& D^{\beta }\overline{\varPhi }_{p}(t_{0}) = \frac{1}{\varGamma (n-\beta )} \biggl[ \int _{0}^{t}(t-\zeta )^{n- \beta -1} \frac{d^{n}}{d\zeta ^{n}}\overline{\varPhi }_{p}(\zeta ) \,d\zeta \biggr]_{t=t_{0}}, \end{aligned}$$

whenever the integrals on the right-hand sides converge and $n=\lceil \beta \rceil $.

### Definition 9

([30, 31, 33])

Let *Φ* be a continuous fuzzy-valued function. Assume that $\varPhi (\chi )\cdot e^{-s\chi }$ is improper fuzzy Riemann-integrable on $[0,\infty )$, then its fuzzy Laplace transform is represented by

$$ \boldsymbol{L}\bigl[\varPhi (\chi )\bigr]= \int _{0}^{\infty }\varPhi (\chi ) \cdot e^{-s\chi }\,d\chi . $$

For $0\leq r\leq 1$, the parametric form of $\varPhi (\chi )$ is represented by

$$ \int _{0}^{\infty }\varPhi (\chi ,r)\cdot e^{-s\chi }\,d\chi = \biggl[ \int _{0}^{\infty }\underline{\varPhi }(\chi ,r)\cdot e^{-s\chi }\,d\chi , \int _{0}^{\infty }\overline{\varPhi }(\chi ,r)\cdot e^{-s\chi }\,d\chi \biggr]. $$

Hence,

$$ \boldsymbol{L}\bigl[\varPhi (\chi ,r)\bigr]= \bigl[\boldsymbol{L} \underline{ \varPhi }(\chi ,r),\boldsymbol{L}\overline{\varPhi }(\chi ,r) \bigr]. $$

### Theorem 1

([31])

*Let* $\varPhi \in C^{F}[0,b]\cap L^{F}[0,b]$, *then for* $0\leq p\leq 1$, *and* $0<\beta \leq 1$, *the Laplace transform of fuzzy fractional derivative in Caputo’s sense is given by*

$$ \boldsymbol{L} \bigl[\bigl(D^{\beta }\varPhi (t)\bigr)_{p} \bigr]=s^{\beta } \boldsymbol{L}\bigl[\varPhi (t)\bigr]-s^{\beta -1} \bigl[\varPhi (0)\bigr]. $$

## Main results

In the following section, the existence and uniqueness of solution to the subsequent fuzzy fractional model are discussed; and we provide the procedure for finding a semianalytic solution of model (2) by using fuzzy Laplace transform.

### Existence and uniqueness

In this section, by the use of fixed point theory, the existence and uniqueness of the subsequent fuzzy fractional model is discussed. Consider the right-hand sides of model (2):

$$\begin{aligned}& \varPsi \bigl(t,\mathcal{Y}_{k}(t),\mathcal{V}_{k}(t), \mathcal{I}_{k}(t), \mathcal{A}_{k}(t), \mathcal{R}_{k}(t),\mathcal{M}_{k}(t)\bigr) \\& \quad = \tilde{n}_{k}-\tilde{m}_{k}\mathcal{Y}_{k}- \tilde{b}_{k}\mathcal{Y}_{k}( \mathcal{I}_{k}+ \tilde{\kappa }\mathcal{A}_{k})-\tilde{b}_{k} \mathcal{Y}_{k}\mathcal{M}_{k}, \\& \varXi \bigl(t,\mathcal{Y}_{k}(t),\mathcal{V}_{k}(t), \mathcal{I}_{k}(t), \mathcal{A}_{k}(t), \mathcal{R}_{k}(t),\mathcal{M}_{k}(t)\bigr) \\& \quad = \bigl\{ \tilde{b}_{k}\mathcal{Y}_{k}(\mathcal{I}_{k}+ \tilde{\delta } \mathcal{A}_{k})+\tilde{b}_{l} \mathcal{Y}_{k}\mathcal{M}_{k}-(1- \tilde{\delta }_{k})\tilde{\omega }_{k}\mathcal{V}_{k} -\tilde{\delta }_{k}\tilde{\omega }_{k}^{\prime } \mathcal{V}_{k}- \tilde{m}_{k}\mathcal{V}_{k} \bigr\} , \\& f\bigl(t,\mathcal{Y}_{k}(t),\mathcal{V}_{k}(t), \mathcal{I}_{k}(t), \mathcal{A}_{k}(t), \mathcal{R}_{k}(t),\mathcal{M}_{k}(t)\bigr) = (1- \tilde{\delta }_{k})\tilde{\omega }_{k} \mathcal{V}_{k}-(\tilde{\gamma }_{k}+ \tilde{m}_{k})\mathcal{I}_{k}, \\& g\bigl(t,\mathcal{Y}_{k}(t),\mathcal{V}_{k}(t), \mathcal{I}_{k}(t), \mathcal{A}_{k}(t), \mathcal{R}_{k}(t),\mathcal{M}_{k}(t)\bigr) = \tilde{\delta }_{k}\tilde{\omega }_{k}^{\prime } \mathcal{V}_{k}-\bigl( \tilde{\gamma }_{k}^{\prime }+ \tilde{m}_{k}\bigr)\mathcal{A}_{k}, \\& h\bigl(t,\mathcal{Y}_{k}(t),\mathcal{V}_{k}(t), \mathcal{I}_{k}(t), \mathcal{A}_{k}(t), \mathcal{R}_{k}(t),\mathcal{M}_{k}(t)\bigr) = \tilde{\gamma }_{k}\mathcal{I}_{k}+\tilde{\gamma }_{k}^{\prime }\mathcal{A}_{k}- \tilde{m}_{k}\mathcal{R}_{k}, \\& y\bigl(t,\mathcal{Y}_{k}(t),\mathcal{V}_{k}(t), \mathcal{I}_{k}(t), \mathcal{A}_{k}(t), \mathcal{R}_{k}(t),\mathcal{M}_{k}(t)\bigr) = \tilde{\xi }\mathcal{I}_{k}+\tilde{\eta }\mathcal{A}_{k}- \tilde{\nu } \mathcal{M}_{k}, \end{aligned}$$

where *Ψ*, *Ξ*, *f*, *g*, *h*, and *y* are fuzzy functions. Thus, for $0<\gamma \leq 1$, the given model (2) can be written as:

3 $$ \textstyle\begin{cases} D_{t}^{\gamma }\mathcal{Y}_{k}(t) =\varPsi (t,\mathcal{Y}_{k}(t), \mathcal{V}_{k}(t),\mathcal{I}_{k}(t),\mathcal{A}_{k}(t),\mathcal{R}_{k}(t), \mathcal{M}_{k}(t)), \\ D_{t}^{\gamma }\mathcal{V}_{k}(t) =\varXi (t,\mathcal{Y}_{k}(t), \mathcal{V}_{k}(t),\mathcal{I}_{k}(t),\mathcal{A}_{k}(t),\mathcal{R}_{k}(t), \mathcal{M}_{k}(t)), \\ D_{t}^{\gamma }\mathcal{I}_{k}(t) =f(t,\mathcal{Y}_{k}(t), \mathcal{V}_{k}(t),\mathcal{I}_{k}(t),\mathcal{A}_{k}(t),\mathcal{R}_{k}(t), \mathcal{M}_{k}(t)), \\ D_{t}^{\gamma }\mathcal{A}_{k}(t) =g(t,\mathcal{Y}_{k}(t), \mathcal{V}_{k}(t),\mathcal{I}_{k}(t),\mathcal{A}_{k}(t),\mathcal{R}_{k}(t), \mathcal{M}_{k}(t)), \\ D_{t}^{\gamma }\mathcal{R}_{k}(t) =h(t,\mathcal{Y}_{k}(t), \mathcal{V}_{k}(t),\mathcal{I}_{k}(t),\mathcal{A}_{k}(t),\mathcal{R}_{k}(t), \mathcal{M}_{k}(t)), \\ D_{t}^{\gamma }\mathcal{M}_{k}(t) =y(t,\mathcal{Y}_{k}(t), \mathcal{V}_{k}(t),\mathcal{I}_{k}(t),\mathcal{A}_{k}(t),\mathcal{R}_{k}(t), \mathcal{M}_{k}(t)), \end{cases} $$

with fuzzy initial conditions

$$\begin{aligned}& \tilde{\mathcal{Y}}(0,\alpha )=\bigl(\underline{\mathcal{Y}}(0,\alpha ), \overline{\mathcal{Y}}(0,\alpha )\bigr), \qquad \tilde{\mathcal{V}}(0,\alpha )=\bigl( \underline{\mathcal{V}}(0,\alpha ),\overline{\mathcal{V}}(0,\alpha )\bigr), \\& \tilde{\mathcal{I}}(0,\alpha )=\bigl(\underline{\mathcal{I}}(0,\alpha ), \overline{\mathcal{I}}(0,\alpha )\bigr), \qquad \tilde{\mathcal{A}}(0,\alpha )=\bigl( \underline{\mathcal{A}}(0,\alpha ),\overline{\mathcal{A}}(0,\alpha )\bigr), \\& \tilde{\mathcal{R}}(0,\alpha )=\bigl(\underline{\mathcal{R}}(0,\alpha ), \overline{\mathcal{R}}(0,\alpha )\bigr), \qquad \tilde{\mathcal{M}}(0,\alpha )=\bigl( \underline{\mathcal{M}}(0,\alpha ),\overline{\mathcal{M}}(0,\alpha )\bigr). \end{aligned}$$

Now applying fuzzy fractional integral $I^{r}$ and using initial conditions, we get

4 $$ \textstyle\begin{cases} \mathcal{Y}_{k}(t) =\tilde{\mathcal{Y}}(0,\alpha )+ \frac{1}{\varGamma (\gamma )}\int _{0}^{t}(t-s)^{\gamma -1}\varPsi (s, \mathcal{Y}_{k}(s),\mathcal{V}_{k}(s),\mathcal{I}_{k}(s),\mathcal{A}_{k}(s), \mathcal{R}_{k}(s),\mathcal{M}_{k}(s))\,ds, \\ \mathcal{V}_{k}(t) =\tilde{\mathcal{V}}(0,\alpha )+ \frac{1}{\varGamma (\gamma )}\int _{0}^{t}(t-s)^{\gamma -1}\varXi (s, \mathcal{Y}_{k}(s),\mathcal{V}_{k}(s),\mathcal{I}_{k}(s),\mathcal{A}_{k}(s), \mathcal{R}_{k}(s),\mathcal{M}_{k}(s))\,ds, \\ \mathcal{I}_{k}(t) =\tilde{\mathcal{I}}(0,\alpha )+ \frac{1}{\varGamma (\gamma )}\int _{0}^{t}(t-s)^{\gamma -1}f(s, \mathcal{Y}_{k}(s),\mathcal{V}_{k}(s),\mathcal{I}_{k}(s),\mathcal{A}_{k}(s), \mathcal{R}_{k}(s),\mathcal{M}_{k}(s))\,ds, \\ \mathcal{A}_{k}(t) =\tilde{\mathcal{A}}(0,\alpha )+ \frac{1}{\varGamma (\gamma )}\int _{0}^{t}(t-s)^{\gamma -1}g(s, \mathcal{Y}_{k}(s),\mathcal{V}_{k}(s),\mathcal{I}_{k}(s),\mathcal{A}_{k}(s), \mathcal{R}_{k}(s),\mathcal{M}_{k}(s))\,ds, \\ \mathcal{R}_{k}(t) =\tilde{\mathcal{R}}(0,\alpha )+ \frac{1}{\varGamma (\gamma )}\int _{0}^{t}(t-s)^{\gamma -1}h(s, \mathcal{Y}_{k}(s),\mathcal{V}_{k}(s),\mathcal{I}_{k}(s),\mathcal{A}_{k}(s), \mathcal{R}_{k}(s),\mathcal{M}_{k}(s))\,ds, \\ \mathcal{M}_{k}(t) =\tilde{\mathcal{M}}(0,\alpha )+ \frac{1}{\varGamma (\gamma )}\int _{0}^{t}(t-s)^{\gamma -1}y(s, \mathcal{Y}_{k}(s),\mathcal{V}_{k}(s),\mathcal{I}_{k}(s),\mathcal{A}_{k}(s), \mathcal{R}_{k}(s),\mathcal{M}_{k}(s))\,ds. \end{cases} $$

Let us define a Banach space as $\mathbb{B}=\mathbb{B}_{1}\times \mathbb{B}_{2}$ under the fuzzy norm:

$$\begin{aligned} &\bigl\Vert \bigl(\mathcal{Y}_{k}(t),\mathcal{V}_{k}(t), \mathcal{I}_{k}(t), \mathcal{A}_{k}(t), \mathcal{R}_{k}(t),\mathcal{M}_{k}(t)\bigr) \bigr\Vert \\ &\quad =\max_{t\in [0,T]} \bigl[ \bigl\vert (\mathcal{Y}_{k}(t)+ \mathcal{V}_{k}(t)+\mathcal{I}_{k}(t)+ \mathcal{A}_{k}(t)+\mathcal{R}_{k}(t)+ \mathcal{M}_{k}(t) \bigr\vert \bigr]. \end{aligned}$$

One can write equation (4) as

5 $$ \tilde{\aleph }_{k}(t)=\tilde{\aleph }(0,\alpha )+ \frac{1}{\varGamma (\gamma )} \int _{0}^{t}(t-\varLambda )^{\gamma -1} \varTheta \bigl(\varLambda ,\tilde{\aleph }_{k}(\varLambda )\bigr)\, d \varLambda , $$

where

$$\begin{aligned}& \tilde{\aleph }_{k}(t)= \textstyle\begin{cases} \mathcal{Y}_{k}(t), \\ \mathcal{V}_{k}(t), \\ \mathcal{I}_{k}(t), \\ \mathcal{A}_{k}(t), \\ \mathcal{R}_{k}(t), \\ \mathcal{M}_{k}(t), \end{cases}\displaystyle \qquad \tilde{\aleph }_{k}(0,\alpha )= \textstyle\begin{cases} \tilde{\mathcal{Y}}(0,\alpha ), \\ \tilde{\mathcal{V}}(0,\alpha ), \\ \tilde{\mathcal{I}}(0,\alpha ), \\ \tilde{\mathcal{A}}(0,\alpha ), \\ \tilde{\mathcal{R}}(0,\alpha ), \\ \tilde{\mathcal{M}}(0,\alpha ), \end{cases}\displaystyle \quad \mbox{and} \\& \varTheta \bigl(t,\tilde{\aleph }_{k}(t)\bigr)= \textstyle\begin{cases} \varPsi (t,\mathcal{Y}_{k}(t),\mathcal{V}_{k}(t),\mathcal{I}_{k}(t), \mathcal{A}_{k}(t),\mathcal{R}_{k}(t),\mathcal{M}_{k}(t)), \\ \varXi (t,\mathcal{Y}_{k}(t),\mathcal{V}_{k}(t),\mathcal{I}_{k}(t), \mathcal{A}_{k}(t),\mathcal{R}_{k}(t),\mathcal{M}_{k}(t)), \\ f(t,\mathcal{Y}_{k}(t),\mathcal{V}_{k}(t),\mathcal{I}_{k}(t), \mathcal{A}_{k}(t),\mathcal{R}_{k}(t),\mathcal{M}_{k}(t)), \\ g(t,\mathcal{Y}_{k}(t),\mathcal{V}_{k}(t),\mathcal{I}_{k}(t), \mathcal{A}_{k}(t),\mathcal{R}_{k}(t),\mathcal{M}_{k}(t)), \\ h(t,\mathcal{Y}_{k}(t),\mathcal{V}_{k}(t),\mathcal{I}_{k}(t), \mathcal{A}_{k}(t),\mathcal{R}_{k}(t),\mathcal{M}_{k}(t)), \\ y(t,\mathcal{Y}_{k}(t),\mathcal{V}_{k}(t),\mathcal{I}_{k}(t), \mathcal{A}_{k}(t),\mathcal{R}_{k}(t),\mathcal{M}_{k}(t)). \end{cases}\displaystyle \end{aligned}$$

We make several assumptions on the nonlinear function $\varTheta :\mathbb{B}\rightarrow \mathbb{B}$ as follows:

1. There exists constant $K_{\aleph }>0$ such that for each $\tilde{\aleph }_{k_{1}}(t),\tilde{\aleph }_{k_{2}}(t)\in \mathbb{B}$,
  $$ \bigl\vert \varTheta \bigl(t,\tilde{\aleph }_{k_{1}}(t)\bigr)-\varTheta \bigl(t,\tilde{\aleph }_{k_{2}}(t)\bigr) \bigr\vert \leq K_{\aleph } \bigl\vert \tilde{\aleph }_{k_{1}}(t)- \tilde{ \aleph }_{k_{2}}(t) \bigr\vert . $$
2. There exist constants $M_{\aleph }>0$ and $N_{\aleph }>0$ such that
  $$ \bigl\vert \varTheta \bigl(t,\tilde{\aleph }_{k}(t)\bigr) \bigr\vert \leq M_{\aleph } \bigl\vert \tilde{\aleph }_{k}(t) \bigr\vert +N_{\aleph }. $$

#### Theorem 2

*Under Assumption* (C-2), *the considered model* (3) *has at least one solution*.

#### Proof

Let $\mathscr{A}= \{ \tilde{\aleph }_{k}(t)\in \mathbb{B}: \Vert \tilde{\aleph }_{k}(t) \Vert \leq \mathsf{r} \} \subset \mathbb{B}$ be a closed and convex fuzzy set, and $\psi :\mathscr{A}\rightarrow \mathscr{A}$ be a mapping defined as

6 $$ \psi \bigl(\tilde{\aleph }_{k}(t)\bigr)=\tilde{\aleph }(0,\alpha )+ \frac{1}{\varGamma (\gamma )} \int _{0}^{t}(t-\varLambda )^{\gamma -1} \varTheta \bigl(\varLambda ,\tilde{\aleph }_{k}(\varLambda )\bigr)\, d \varLambda . $$

For any $\tilde{\aleph _{k}}(t)\in \mathscr{A}$, we have

$$\begin{aligned} \bigl\Vert \psi \bigl(\tilde{\aleph _{k}}(t)\bigr) \bigr\Vert = & \max_{t\in [0,T]} \biggl\vert \tilde{\aleph }(0,\alpha )+ \frac{1}{\varGamma (\gamma )} \int _{0}^{t}(t-\varLambda )^{\gamma -1} \varTheta \bigl(\varLambda ,\tilde{\aleph }_{k}(\varLambda )\bigr)\, d \varLambda \biggr\vert \\ \leq & \bigl\vert \tilde{\aleph }(0,\alpha ) \bigr\vert + \frac{1}{\varGamma (\gamma )} \int _{0}^{t}(t-\varLambda )^{\gamma -1} \bigl\vert \varTheta \bigl(\varLambda ,\tilde{\aleph }_{k}(\varLambda )\bigr) \bigr\vert \, d \varLambda \\ \leq & \bigl\vert \tilde{\aleph }(0,\alpha ) \bigr\vert + \frac{1}{\varGamma (\gamma )} \int _{0}^{t}(t-\varLambda )^{\gamma -1} \bigl[M_{\aleph } \bigl\vert \tilde{\aleph }_{k}(t) \bigr\vert +N_{\aleph } \bigr]\,d\varLambda \\ \leq & \bigl\vert \tilde{\aleph }(0,\alpha ) \bigr\vert + \frac{\tau ^{\gamma }}{\varGamma (\gamma +1)} \bigl[M_{\aleph } \bigl\vert \tilde{\aleph }_{k}(t) \bigr\vert +N_{\aleph } \bigr]. \end{aligned}$$

From the last inequality, we have $\psi (\mathscr{A})\subset \mathscr{A}$, which implies that the operator *ψ* is bounded. Next we show that the operator *ψ* is completely continuous. For this, let $\phi _{1},\phi _{2}\in [0,T]$ be such that $\phi _{1}<\phi _{2}$, then

$$\begin{aligned} \bigl\Vert \psi \bigl(\tilde{\aleph }_{k}(t)\bigr) (\phi _{2})-\psi \bigl( \tilde{\aleph }_{k}(t)\bigr) (\phi _{1}) \bigr\Vert = & \biggl\vert \frac{1}{\varGamma (\gamma )} \int _{0}^{\phi _{2}}(\phi _{2}- \varLambda )^{\gamma -1}\varTheta \bigl(\varLambda ,\tilde{\aleph }_{k}( \varLambda )\bigr)\,d\varLambda \\ &{} -\frac{1}{\varGamma (\gamma )} \int _{0}^{\phi _{1}}(\phi _{1}- \varLambda )^{\gamma -1}\varTheta \bigl(\varLambda ,\tilde{\aleph }_{k}( \varLambda )\bigr)\,d\varLambda \biggr\vert \\ \leq & \bigl[\phi _{2}^{\gamma }-\phi _{1}^{\gamma } \bigr] \frac{ [M_{\aleph } \vert \tilde{\aleph }_{k}(t) \vert +N_{\aleph } ]}{\varGamma (\gamma +1)}. \end{aligned}$$

From the last inequality, we see that the right-hand side goes to zero as $\phi _{2}\rightarrow \phi _{1}$. Hence,

$$ \bigl\Vert \psi \bigl(\tilde{\aleph }_{k}(t)\bigr) (\phi _{2})-\psi \bigl( \tilde{\aleph }_{k}(t)\bigr) (\phi _{1}) \bigr\Vert \rightarrow 0 \quad \text{as } \phi _{2}\rightarrow \phi _{1}. $$

Thus, the operator *ψ* is equicontinuous. By Arzela–Ascoli theorem, the operator *ψ* is completely continuous, also *ψ* is bounded as proved earlier. Therefore, system (3) has at least one solution by Schauder’s fixed point theorem. □

#### Theorem 3

*If Assumption* (C-1) *holds*, *then the considered system* (3) *has a unique solution if* $\tau ^{\gamma }K_{\aleph }<\varGamma (\gamma +1)$.

#### Proof

Let $\tilde{\aleph }_{k_{1}}(t),\tilde{\aleph }_{k_{2}}(t)\in \mathbb{B}$, then

$$\begin{aligned} \bigl\Vert \psi \bigl(\tilde{\aleph }_{k_{1}}(t)\bigr)-\psi \bigl( \tilde{\aleph }_{k_{2}}(t)\bigr) \bigr\Vert = & \max _{t\in [0,T]} \biggl\vert \frac{1}{\varGamma (\gamma )} \int _{0}^{t}(t-\varLambda )^{\gamma -1} \varTheta \bigl(\varLambda ,\tilde{\aleph }_{k_{1}}(\varLambda )\bigr)\, d \varLambda \\ &{} -\frac{1}{\varGamma (\gamma )} \int _{0}^{t}(t-\varLambda )^{ \gamma -1} \varTheta \bigl(\varLambda ,\tilde{\aleph }_{k_{2}}(\varLambda )\bigr)\, d \varLambda \biggr\vert \\ \leq & \frac{\tau ^{\gamma }}{\varGamma (\gamma +1)}K_{\aleph } \bigl\vert \tilde{\aleph }_{k_{1}}(t)-\tilde{\aleph }_{k_{2}}(t) \bigr\vert . \end{aligned}$$

Hence *ψ* is a contraction. Hence, by Banach contraction theorem, system (3) has a unique solution. □

### Procedure for solution

Here a general method is provided in order to find the solution of the considered system by the fuzzy Laplace transform.

Taking fuzzy Laplace transform of (3) and using initial conditions, we get

$$\begin{aligned}& \boldsymbol{L} \bigl[D_{t}^{\gamma }\bigl[ \mathcal{Y}_{k}(t)\bigr] \bigr] = \boldsymbol{L} \bigl[\varPsi \bigl(t,\mathcal{Y}_{k}(t),\mathcal{V}_{k}(t), \mathcal{I}_{k}(t),\mathcal{A}_{k}(t), \mathcal{R}_{k}(t),\mathcal{M}_{k}(t)\bigr) \bigr], \\& \boldsymbol{L} \bigl[D_{t}^{\gamma }\bigl[ \mathcal{V}_{k}(t)\bigr] \bigr] = \boldsymbol{L} \bigl[\varXi \bigl(t,\mathcal{Y}_{k}(t),\mathcal{V}_{k}(t), \mathcal{I}_{k}(t),\mathcal{A}_{k}(t), \mathcal{R}_{k}(t),\mathcal{M}_{k}(t)\bigr) \bigr], \\& \boldsymbol{L} \bigl[D_{t}^{\gamma }\bigl[ \mathcal{I}_{k}(t)\bigr] \bigr] = \boldsymbol{L} \bigl[f\bigl(t, \mathcal{Y}_{k}(t),\mathcal{V}_{k}(t), \mathcal{I}_{k}(t),\mathcal{A}_{k}(t), \mathcal{R}_{k}(t),\mathcal{M}_{k}(t)\bigr) \bigr], \\& \boldsymbol{L} \bigl[D_{t}^{\gamma }\bigl[ \mathcal{A}_{k}(t)\bigr] \bigr] = \boldsymbol{L} \bigl[g\bigl(t, \mathcal{Y}_{k}(t),\mathcal{V}_{k}(t), \mathcal{I}_{k}(t),\mathcal{A}_{k}(t), \mathcal{R}_{k}(t),\mathcal{M}_{k}(t)\bigr) \bigr], \\& \boldsymbol{L} \bigl[D_{t}^{\gamma }\bigl[ \mathcal{R}_{k}(t)\bigr] \bigr] = \boldsymbol{L} \bigl[h\bigl(t, \mathcal{Y}_{k}(t),\mathcal{V}_{k}(t), \mathcal{I}_{k}(t),\mathcal{A}_{k}(t), \mathcal{R}_{k}(t),\mathcal{M}_{k}(t)\bigr) \bigr], \\& \boldsymbol{L} \bigl[D_{t}^{\gamma }\bigl[ \mathcal{M}_{k}(t)\bigr] \bigr] = \boldsymbol{L} \bigl[y\bigl(t, \mathcal{Y}_{k}(t),\mathcal{V}_{k}(t), \mathcal{I}_{k}(t),\mathcal{A}_{k}(t), \mathcal{R}_{k}(t),\mathcal{M}_{k}(t)\bigr) \bigr], \\& s^{\gamma }\boldsymbol{L}\bigl[\mathcal{Y}_{k}(t)\bigr] = s^{\gamma -1} \tilde{\mathcal{Y}}(0,\alpha )+\boldsymbol{L} \bigl[\varPsi \bigl(t, \mathcal{Y}_{k}(t),\mathcal{V}_{k}(t), \mathcal{I}_{k}(t),\mathcal{A}_{k}(t), \mathcal{R}_{k}(t),\mathcal{M}_{k}(t)\bigr) \bigr], \\& s^{\gamma }\boldsymbol{L}\bigl[\mathcal{V}_{k}(t)\bigr] = s^{\gamma -1} \tilde{\mathcal{V}}(0,\alpha )+\boldsymbol{L} \bigl[\varXi \bigl(t, \mathcal{Y}_{k}(t),\mathcal{V}_{k}(t), \mathcal{I}_{k}(t),\mathcal{A}_{k}(t), \mathcal{R}_{k}(t),\mathcal{M}_{k}(t)\bigr) \bigr], \\& s^{\gamma }\boldsymbol{L}\bigl[\mathcal{I}_{k}(t)\bigr] = s^{\gamma -1} \tilde{\mathcal{I}}(0,\alpha )+\boldsymbol{L} \bigl[f\bigl(t, \mathcal{Y}_{k}(t), \mathcal{V}_{k}(t), \mathcal{I}_{k}(t),\mathcal{A}_{k}(t), \mathcal{R}_{k}(t), \mathcal{M}_{k}(t)\bigr) \bigr], \\& s^{\gamma }\boldsymbol{L}\bigl[\mathcal{A}_{k}(t)\bigr] = s^{\gamma -1} \tilde{\mathcal{A}}(0,\alpha )+\boldsymbol{L} \bigl[g\bigl(t, \mathcal{Y}_{k}(t), \mathcal{V}_{k}(t), \mathcal{I}_{k}(t),\mathcal{A}_{k}(t), \mathcal{R}_{k}(t), \mathcal{M}_{k}(t)\bigr) \bigr], \\& s^{\gamma }\boldsymbol{L}\bigl[\mathcal{R}_{k}(t)\bigr] = s^{\gamma -1} \tilde{\mathcal{R}}(0,\alpha )+\boldsymbol{L} \bigl[h\bigl(t, \mathcal{Y}_{k}(t), \mathcal{V}_{k}(t), \mathcal{I}_{k}(t),\mathcal{A}_{k}(t), \mathcal{R}_{k}(t), \mathcal{M}_{k}(t)\bigr) \bigr], \\& s^{\gamma }\boldsymbol{L}\bigl[\mathcal{M}_{k}(t)\bigr] = s^{\gamma -1} \tilde{\mathcal{M}}(0,\alpha )+\boldsymbol{L} \bigl[y\bigl(t, \mathcal{Y}_{k}(t), \mathcal{V}_{k}(t), \mathcal{I}_{k}(t),\mathcal{A}_{k}(t), \mathcal{R}_{k}(t), \mathcal{M}_{k}(t)\bigr) \bigr], \\& \boldsymbol{L}\bigl[\mathcal{Y}_{k}(t)\bigr] = \frac{1}{s} \tilde{\mathcal{Y}}(0,\alpha )+\frac{1}{s^{\gamma }} \boldsymbol{L} \bigl[\varPsi \bigl(t,\mathcal{Y}_{k}(t), \mathcal{V}_{k}(t),\mathcal{I}_{k}(t), \mathcal{A}_{k}(t),\mathcal{R}_{k}(t), \mathcal{M}_{k}(t)\bigr) \bigr], \\& \boldsymbol{L}\bigl[\mathcal{V}_{k}(t)\bigr] = \frac{1}{s} \tilde{\mathcal{V}}(0,\alpha )+\frac{1}{s^{\gamma }} \boldsymbol{L} \bigl[\varXi \bigl(t,\mathcal{Y}_{k}(t), \mathcal{V}_{k}(t),\mathcal{I}_{k}(t), \mathcal{A}_{k}(t),\mathcal{R}_{k}(t), \mathcal{M}_{k}(t)\bigr) \bigr], \\& \boldsymbol{L}\bigl[\mathcal{I}_{k}(t)\bigr] = \frac{1}{s} \tilde{\mathcal{I}}(0,\alpha )+\frac{1}{s^{\gamma }} \boldsymbol{L} \bigl[f\bigl(t,\mathcal{Y}_{k}(t), \mathcal{V}_{k}(t),\mathcal{I}_{k}(t), \mathcal{A}_{k}(t),\mathcal{R}_{k}(t), \mathcal{M}_{k}(t)\bigr) \bigr], \\& \boldsymbol{L}\bigl[\mathcal{A}_{k}(t)\bigr] = \frac{1}{s} \tilde{\mathcal{A}}(0,\alpha )+\frac{1}{s^{\gamma }} \boldsymbol{L} \bigl[g\bigl(t,\mathcal{Y}_{k}(t), \mathcal{V}_{k}(t),\mathcal{I}_{k}(t), \mathcal{A}_{k}(t),\mathcal{R}_{k}(t), \mathcal{M}_{k}(t)\bigr) \bigr], \\& \boldsymbol{L}\bigl[\mathcal{R}_{k}(t)\bigr] = \frac{1}{s} \tilde{\mathcal{R}}(0,\alpha )+\frac{1}{s^{\gamma }} \boldsymbol{L} \bigl[h\bigl(t,\mathcal{Y}_{k}(t), \mathcal{V}_{k}(t),\mathcal{I}_{k}(t), \mathcal{A}_{k}(t),\mathcal{R}_{k}(t), \mathcal{M}_{k}(t)\bigr) \bigr], \\& \boldsymbol{L}\bigl[\mathcal{M}_{k}(t)\bigr] = \frac{1}{s} \tilde{\mathcal{M}}(0,\alpha )+\frac{1}{s^{\gamma }} \boldsymbol{L} \bigl[y\bigl(t,\mathcal{Y}_{k}(t), \mathcal{V}_{k}(t),\mathcal{I}_{k}(t), \mathcal{A}_{k}(t),\mathcal{R}_{k}(t), \mathcal{M}_{k}(t)\bigr) \bigr]. \end{aligned}$$

The infinite series solution is given by:

$$\begin{aligned}& \mathcal{Y}_{k}(t)=\sum_{n=0}^{\infty } \mathcal{Y}_{k_{n}}(t), \qquad \mathcal{V}_{k}(t)=\sum _{n=0}^{\infty }\mathcal{V}_{k_{n}}(t), \\& \mathcal{I}_{k}(t)=\sum_{n=0}^{\infty } \mathcal{I}_{k_{n}}(t) , \qquad \mathcal{A}_{k}(t)=\sum _{n=0}^{\infty }\mathcal{A}_{k_{n}}(t), \\& \mathcal{R}_{k}(t)=\sum_{n=0}^{\infty } \mathcal{R}_{k_{n}}(t) , \qquad \mathcal{M}_{k}(\text{$t$})= \sum_{n=0}^{\infty }\mathcal{M}_{k_{n}}(t), \\& \mathcal{Y}_{k}(t)\mathcal{I}_{k}(t) = \sum _{n=0}^{\infty } \mathcal{Z}_{1,n}, \\& \mathcal{Y}_{k}(t)\mathcal{A}_{k}(t) = \sum _{n=0}^{\infty } \mathcal{Z}_{2,n}, \\& \mathcal{Y}_{k}(t)\mathcal{M}_{k}(t) = \sum _{n=0}^{\infty } \mathcal{Z}_{3,n}, \end{aligned}$$

where $\mathcal{Z}_{1_{n}}$, $\mathcal{Z}_{2_{n}}$, and $\mathcal{Z}_{3_{n}}$ are Adomian polynomials, representing nonlinear terms. So the last equation becomes

$$\begin{aligned}& \boldsymbol{L} \Biggl[\sum_{n=0}^{\infty } \mathcal{Y}_{k_{n}}(t) \Biggr] = \frac{1}{s}\tilde{ \mathcal{Y}}(0,\alpha )+ \frac{1}{s^{\gamma }}\boldsymbol{L} \bigl[\varPsi \bigl(t, \mathcal{Y}_{k}(t), \mathcal{V}_{k}(t), \mathcal{I}_{k}(t),\mathcal{A}_{k}(t), \mathcal{R}_{k}(t), \mathcal{M}_{k}(t)\bigr) \bigr], \\& \boldsymbol{L} \Biggl[\sum_{n=0}^{\infty } \mathcal{V}_{k_{n}}(t) \Biggr] = \frac{1}{s}\tilde{ \mathcal{V}}(0,\alpha )+ \frac{1}{s^{\gamma }}\boldsymbol{L} \bigl[\varXi \bigl(t, \mathcal{Y}_{k}(t), \mathcal{V}_{k}(t), \mathcal{I}_{k}(t),\mathcal{A}_{k}(t), \mathcal{R}_{k}(t), \mathcal{M}_{k}(t)\bigr) \bigr], \\& \boldsymbol{L} \Biggl[\sum_{n=0}^{\infty } \mathcal{I}_{k_{n}}(t) \Biggr] = \frac{1}{s}\tilde{ \mathcal{I}}(0,\alpha )+ \frac{1}{s^{\gamma }}\boldsymbol{L} \bigl[f\bigl(t, \mathcal{Y}_{k}(t), \mathcal{V}_{k}(t), \mathcal{I}_{k}(t),\mathcal{A}_{k}(t), \mathcal{R}_{k}(t), \mathcal{M}_{k}(t)\bigr) \bigr], \\& \boldsymbol{L} \Biggl[\sum_{n=0}^{\infty } \mathcal{A}_{k_{n}}(t) \Biggr] = \frac{1}{s}\tilde{ \mathcal{A}}(0,\alpha )+ \frac{1}{s^{\gamma }}\boldsymbol{L} \bigl[g\bigl(t, \mathcal{Y}_{k}(t), \mathcal{V}_{k}(t), \mathcal{I}_{k}(t),\mathcal{A}_{k}(t), \mathcal{R}_{k}(t), \mathcal{M}_{k}(t)\bigr) \bigr], \\& \boldsymbol{L} \Biggl[\sum_{n=0}^{\infty } \mathcal{R}_{k_{n}}(t) \Biggr] = \frac{1}{s}\tilde{ \mathcal{R}}(0,\alpha )+ \frac{1}{s^{\gamma }}\boldsymbol{L} \bigl[h\bigl(t, \mathcal{Y}_{k}(t), \mathcal{V}_{k}(t), \mathcal{I}_{k}(t),\mathcal{A}_{k}(t), \mathcal{R}_{k}(t), \mathcal{M}_{k}(t)\bigr) \bigr], \\& \boldsymbol{L} \Biggl[\sum_{n=0}^{\infty } \mathcal{M}_{k_{n}}(t) \Biggr] = \frac{1}{s}\tilde{ \mathcal{M}}(0,\alpha )+ \frac{1}{s^{\gamma }}\boldsymbol{L} \bigl[y\bigl(t, \mathcal{Y}_{k}(t), \mathcal{V}_{k}(t), \mathcal{I}_{k}(t),\mathcal{A}_{k}(t), \mathcal{R}_{k}(t), \mathcal{M}_{k}(t)\bigr) \bigr]. \end{aligned}$$

Taking the inverse Laplace transform, we have

$$\begin{aligned}& \sum_{n=0}^{\infty }\mathcal{Y}_{k_{n}}(t) = \tilde{\mathcal{Y}}(0, \alpha )+\boldsymbol{L^{-1}} \biggl[ \frac{1}{s^{\gamma }}\boldsymbol{L} \bigl[\varPsi \bigl(t,\mathcal{Y}_{k}(t), \mathcal{V}_{k}(t),\mathcal{I}_{k}(t), \mathcal{A}_{k}(t),\mathcal{R}_{k}(t), \mathcal{M}_{k}(t)\bigr) \bigr] \biggr], \\& \sum_{n=0}^{\infty }\mathcal{V}_{k_{n}}(t) = \tilde{\mathcal{V}}(0, \alpha )+\boldsymbol{L^{-1}} \biggl[ \frac{1}{s^{\gamma }}\boldsymbol{L} \bigl[\varXi \bigl(t,\mathcal{Y}_{k}(t), \mathcal{V}_{k}(t),\mathcal{I}_{k}(t), \mathcal{A}_{k}(t),\mathcal{R}_{k}(t), \mathcal{M}_{k}(t)\bigr) \bigr] \biggr], \\& \sum_{n=0}^{\infty }\mathcal{I}_{k_{n}}(t) = \tilde{\mathcal{I}}(0, \alpha )+\boldsymbol{L^{-1}} \biggl[ \frac{1}{s^{\gamma }}\boldsymbol{L} \bigl[f\bigl(t,\mathcal{Y}_{k}(t), \mathcal{V}_{k}(t),\mathcal{I}_{k}(t), \mathcal{A}_{k}(t),\mathcal{R}_{k}(t), \mathcal{M}_{k}(t)\bigr) \bigr] \biggr], \\& \sum_{n=0}^{\infty }\mathcal{A}_{k_{n}}(t) = \tilde{\mathcal{A}}(0, \alpha )+\boldsymbol{L^{-1}} \biggl[ \frac{1}{s^{\gamma }}\boldsymbol{L} \bigl[g\bigl(t,\mathcal{Y}_{k}(t), \mathcal{V}_{k}(t),\mathcal{I}_{k}(t), \mathcal{A}_{k}(t),\mathcal{R}_{k}(t), \mathcal{M}_{k}(t)\bigr) \bigr] \biggr], \\& \sum_{n=0}^{\infty }\mathcal{R}_{k_{n}}(t) = \tilde{\mathcal{R}}(0, \alpha )+\boldsymbol{L^{-1}} \biggl[ \frac{1}{s^{\gamma }}\boldsymbol{L} \bigl[h\bigl(t,\mathcal{Y}_{k}(t), \mathcal{V}_{k}(t),\mathcal{I}_{k}(t), \mathcal{A}_{k}(t),\mathcal{R}_{k}(t), \mathcal{M}_{k}(t)\bigr) \bigr] \biggr], \\& \sum_{n=0}^{\infty }\mathcal{M}_{k_{n}}(t) = \tilde{\mathcal{M}}(0, \alpha )+\boldsymbol{L^{-1}} \biggl[ \frac{1}{s^{\gamma }}\boldsymbol{L} \bigl[y\bigl(t,\mathcal{Y}_{k}(t), \mathcal{V}_{k}(t),\mathcal{I}_{k}(t), \mathcal{A}_{k}(t),\mathcal{R}_{k}(t), \mathcal{M}_{k}(t)\bigr) \bigr] \biggr]. \end{aligned}$$

Comparing the terms on both sides, we consider the first two terms of the series

7 $$\begin{aligned}& \textstyle\begin{cases} \underline{\mathcal{Y}}_{k_{0}}(t)=\underline{\mathcal{Y}}(0,\alpha ), \qquad \overline{S}_{k_{0}}(t)=\overline{\mathcal{Y}}(0,\alpha ), \\ \underline{\mathcal{V}}_{k_{0}}(t)=\underline{\mathcal{V}}(0,\alpha ), \qquad \overline{\mathcal{V}}_{k_{0}}(t)=\overline{\mathcal{V}}(0,\alpha ), \\ \underline{\mathcal{I}}_{k_{0}}(t)=\underline{\mathcal{I}}(0,\alpha ), \qquad \overline{\mathcal{I}}_{k_{0}}(t)=\overline{\mathcal{V}}(0,\alpha ), \\ \underline{\mathcal{A}}_{k_{0}}(t)=\underline{\mathcal{A}}(0,\alpha ), \qquad \overline{\mathcal{A}}_{k_{0}}(t)=\overline{\mathcal{A}}(0,\alpha ), \\ \underline{\mathcal{R}}_{k_{0}}(t)=\underline{\mathcal{R}}(0,\alpha ), \qquad \overline{\mathcal{R}}_{k_{0}}(t)=\overline{\mathcal{R}}(0,\alpha ), \\ \underline{\mathcal{M}}_{k_{0}}(t)=\underline{\mathcal{M}}(0,\alpha ), \qquad \overline{\mathcal{M}}_{k_{0}}(t)=\overline{\mathcal{M}}(0,\alpha ), \end{cases}\displaystyle \end{aligned}$$

8 $$\begin{aligned}& \textstyle\begin{cases} \underline{\mathcal{Y}}_{k_{1}}(t)= \boldsymbol{L^{-1}} [ \frac{1}{s^{\gamma }}\boldsymbol{L} [\tilde{n}_{k}-\tilde{m}_{k} \underline{\mathcal{Y}}_{k_{0}}-\tilde{b}_{k}\underline{\mathcal{Y}}_{k_{0}}( \underline{\mathcal{I}}_{k_{0}}+\tilde{\kappa }\underline{\mathcal{A}}_{k_{0}})- \tilde{b}_{k}\underline{\mathcal{Y}}_{k_{0}}\underline{\mathcal{M}}_{k_{0}} ] ], \\ \overline{\mathcal{Y}}_{k_{1}}(t)= \boldsymbol{L^{-1}} [ \frac{1}{s^{\gamma }}\boldsymbol{L} [\tilde{n}_{k}-\tilde{m}_{k} \overline{\mathcal{Y}}_{k_{1}}-\tilde{b}_{k}\overline{\mathcal{Y}}_{k_{0}}( \overline{\mathcal{I}}_{k_{1}}+\tilde{\kappa }\overline{\mathcal{A}}_{k_{0}})- \tilde{b}_{k}\overline{\mathcal{Y}}_{k_{0}}\overline{\mathcal{M}}_{k_{0}} ] ]. \end{cases}\displaystyle \end{aligned}$$

Similarly, we can find the other terms.

Hence, the series solution of the considered system is given by

9 $$ \textstyle\begin{cases} \underline{\mathcal{Y}}_{k}(t) =\underline{\mathcal{Y}}_{k_{o}}(t)+ \underline{\mathcal{Y}}_{k_{1}}(t)+\underline{\mathcal{Y}}_{k_{2}}(t)+ \cdots , \\ \overline{\mathcal{Y}}_{k}(t) =\overline{\mathcal{Y}}_{k_{0}}(t)+ \overline{\mathcal{Y}}_{k_{1}}(t)+\overline{\mathcal{Y}}_{k_{2}}(t)+ \cdots , \\ \underline{\mathcal{V}}_{k}(t) =\underline{\mathcal{V}}_{k_{o}}(t)+ \underline{\mathcal{V}}_{k_{1}}(t)+\underline{\mathcal{V}}_{k_{2}}(t)+ \cdots , \\ \overline{\mathcal{V}}_{k}(t) =\overline{\mathcal{V}}_{k_{0}}(t)+ \overline{\mathcal{V}}_{k_{1}}(t)+\overline{\mathcal{V}}_{k_{2}}(t)+ \cdots , \\ \underline{\mathcal{I}}_{k}(t) =\underline{\mathcal{I}}_{k_{o}}(t)+ \underline{\mathcal{I}}_{k_{1}}(t)+\underline{\mathcal{I}}_{k_{2}}(t)+ \cdots , \\ \overline{\mathcal{I}}_{k}(t) =\overline{\mathcal{I}}_{k_{0}}(t)+ \overline{\mathcal{I}}_{k_{1}}(t)+\overline{\mathcal{I}}_{k_{2}}(t)+ \cdots , \\ \underline{\mathcal{A}}_{k}(t) =\underline{\mathcal{A}}_{k_{o}}(t)+ \underline{\mathcal{A}}_{k_{1}}(t)+\underline{\mathcal{A}}_{k_{2}}(t)+ \cdots , \\ \overline{\mathcal{A}}_{k}(t) =\overline{\mathcal{A}}_{k_{0}}(t)+ \overline{\mathcal{A}}_{k_{1}}(t)+\overline{\mathcal{A}}_{k_{2}}(t)+ \cdots , \\ \underline{\mathcal{R}}_{k}(t) =\underline{\mathcal{R}}_{k_{o}}(t)+ \underline{\mathcal{R}}_{k_{1}}(t)+\underline{\mathcal{R}}_{k_{2}}(t)+ \cdots , \\ \overline{\mathcal{R}}_{k}(t) =\overline{\mathcal{R}}_{k_{0}}(t)+ \overline{\mathcal{R}}_{k_{1}}(t)+\overline{\mathcal{R}}_{k_{2}}(t)+ \cdots , \\ \underline{\mathcal{M}}_{k}(t) =\underline{\mathcal{M}}_{k_{o}}(t)+ \underline{\mathcal{M}}_{k_{1}}(t)+\underline{\mathcal{M}}_{k_{2}}(t)+ \cdots , \\ \overline{\mathcal{M}}_{k}(t) =\overline{\mathcal{M}}_{k_{0}}(t)+ \overline{\mathcal{M}}_{k_{1}}(t)+\overline{\mathcal{M}}_{k_{2}}(t)+ \cdots .\end{cases} $$

## Numerical results and discussion

We consider a table corresponding to the parameters involved in the model.

**Table 1:** Description of the parameters used in model (2)

Notation	Parameters description	Numerical value
$\tilde{n}_{k}$	Birth rate	1
$\tilde{m}_{k}$	Death rate of infected population	$\frac{1}{(76.79\times 365)}$
$\tilde{b}_{k}$	Transmission coefficient	0.05
$\tilde{b}_{l}$	Disease transmission coefficient	0.001231
$\tilde{\omega }_{k}$, $\tilde{\omega }'_{k}$	Signified incubation period	0.001243, 0.05
$\tilde{\gamma }_{k}$, $\tilde{\gamma }'_{k}$	Recovery rate of ${\mathcal{I}}_{k}$, ${\mathcal{A}}_{k}$	0.09871, 0.0854302
*ξ̃*, *η̃*	Influence of virus from ${\mathcal{I}}_{k}$ and ${\mathcal{A}}_{k}$ to ${\mathcal{M}}_{k}$	0.0398, 0.01
*ν̃*	Amount of asymptotic infection	0.1243
*κ̃*	Transmissibility multiple	0.02
*η̃*	Elimination rate of virus from ${\mathcal{M}}_{k}$	0.01
$\mathcal{Y}_{0}$	Initial value of susceptible	220 million
$\mathcal{I}_{0}$	Initial value of infected	0.015 million
$\mathcal{V}_{0}$	Initial value of exposed	100 million
$\mathcal{A}_{0}$	Initial value of asymptotically infected	0.60 million
$\mathcal{R}_{0}$	Initial value of recovered	0 million
$\mathcal{M}_{0}$	Initial value of reservoir	0.1 million

Consider the proposed model (2) with initial conditions as given in Table 1:

$$\begin{aligned}& \widetilde{\mathcal{Y}}(0,\alpha ) =(2\alpha -1,1-2\alpha ), \qquad \widetilde{ \mathcal{V}}(0,\alpha )=(2\alpha -1,1-2\alpha ), \\& \widetilde{\mathcal{I}}(0,\alpha )=(2\alpha -1,1-2\alpha ),\qquad \widetilde{\mathcal{A}}(0,\alpha ) =(2\alpha -1,1-2\alpha ), \\& \widetilde{ \mathcal{R}}(0,\alpha )=(2\alpha -1,1-2\alpha ),\qquad \widetilde{\mathcal{M}}(0,\alpha )=(2\alpha -1,1-2\alpha ). \end{aligned}$$

Applying the proposed procedure to (2) and using initial conditions, we have

$$\begin{aligned}& \underline{\mathcal{Y}}_{k_{0}}(t,\alpha )=2\alpha -1, \qquad \overline{ \mathcal{Y}}_{p_{0}}(t,\alpha )=1-2\alpha , \\& \underline{\mathcal{V}}_{k_{0}}(t,\alpha )=2\alpha -1, \qquad \overline{ \mathcal{V}}_{p_{0}}(t,\alpha )=1-2\alpha , \\& \underline{\mathcal{I}}_{k_{0}}(t,\alpha )=1-2\alpha , \qquad \overline{ \mathcal{I}}_{p_{0}}(t,\alpha )=1-2\alpha , \\& \underline{\mathcal{A}}_{k_{0}}(t,\alpha )=1-2\alpha , \qquad \overline{ \mathcal{A}}_{p_{0}}(t,\alpha )=1-2\alpha , \\& \underline{\mathcal{R}}_{k_{0}}(t,\alpha )=1-2\alpha , \qquad \overline{ \mathcal{R}}_{p_{0}}(t,\alpha )=1-2\alpha , \\& \underline{\mathcal{M}}_{k_{0}}(t,\alpha )=1-2\alpha , \qquad \overline{ \mathcal{M}}_{p_{0}}(t,\alpha )=1-2\alpha . \end{aligned}$$

The second term of the series solution is

10 $$\begin{aligned}& \textstyle\begin{cases} \underline{\mathcal{Y}}_{k_{1}}(t,\alpha )= [\tilde{n}_{k}- \tilde{m}_{k}(2\alpha -1)-\tilde{b}_{k}(2\alpha -1)^{2} \\ \hphantom{\underline{\mathcal{Y}}_{k_{1}}(t,\alpha )= {}}{} -\kappa \tilde{b}_{k}(2\alpha -1)^{2}-\tilde{b}_{k}(2\alpha -1)^{2}] \frac{t^{\gamma }}{\varGamma (\gamma +1)} ], \\ \overline{\mathcal{Y}}_{k_{1}}(t,\alpha )= [\tilde{n}_{k}- \tilde{m}_{k}(1-2\alpha )-\tilde{b}_{k}(1-2\alpha )^{2} \\ \hphantom{\overline{\mathcal{Y}}_{k_{1}}(t,\alpha )={}}{} -\kappa \tilde{b}_{k}(1-2\alpha )^{2}-\tilde{b}_{k}(1-2 \alpha )]\frac{t^{\gamma }}{\varGamma (\gamma +1)} ], \end{cases}\displaystyle \end{aligned}$$

11 $$\begin{aligned}& \textstyle\begin{cases} \underline{\mathcal{V}}_{k_{1}}(t,\alpha )= [\tilde{b}_{k}(2 \alpha -1)^{2}+\kappa \tilde{b}_{k}(2\alpha -1)^{2}+\tilde{b}_{k}(2 \alpha -1)^{2}-(1-\tilde{\delta }_{k})\tilde{\omega }_{k}(2\alpha -1) \\ \hphantom{\underline{\mathcal{V}}_{k_{1}}(t,\alpha )={}}{} -\tilde{\delta }_{k}\tilde{\omega }_{k}^{\prime }(2\alpha -1)- \tilde{m}_{k}(2\alpha -1)]\frac{t^{\gamma }}{\varGamma (\gamma +1)} ], \\ \overline{\mathcal{V}}_{k_{1}}(t,\alpha )= [\tilde{b}_{k}(1-2 \alpha )^{2}+\kappa \tilde{b}_{k}(1-2\alpha )^{2}+\tilde{b}_{k}(1-2 \alpha )^{2}-(1-\tilde{\delta }_{k})\tilde{\omega }_{k}(1-2\alpha ) \\ \hphantom{\overline{\mathcal{V}}_{k_{1}}(t,\alpha )={}}{} -\tilde{\delta }_{k}\tilde{\omega }_{k}^{\prime }(1-2\alpha )- \tilde{m}_{k}(1-2\alpha )]\frac{t^{\gamma }}{\varGamma (\gamma +1)} ], \end{cases}\displaystyle \end{aligned}$$

12 $$\begin{aligned}& \textstyle\begin{cases} \underline{\mathcal{I}}_{k_{1}}(t,\alpha )= [(1- \tilde{\delta }_{k})\tilde{\omega }_{k}(2\alpha -1)-(\hat{\gamma }_{p}+ \tilde{m}_{k})(2\alpha -1) ] \frac{t^{\gamma }}{\varGamma (\gamma +1)}, \\ \overline{\mathcal{I}}_{k_{1}}(t,\alpha )= [(1-\tilde{\delta }_{k}) \tilde{\omega }_{k}(1-2\alpha )-(\hat{\gamma }_{p}+\tilde{m}_{k})(1-2 \alpha ) ]\frac{t^{\gamma }}{\varGamma (\gamma +1)}, \end{cases}\displaystyle \end{aligned}$$

13 $$\begin{aligned}& \textstyle\begin{cases} \underline{\mathcal{A}}_{k_{1}}(t,\alpha )= [\tilde{\delta }_{k} \tilde{\omega }_{k}^{\prime }(2\alpha -1)-(\hat{\gamma }_{k}^{\prime }+\tilde{m}_{k})(2 \alpha -1) ]\frac{t^{\gamma }}{\varGamma (\gamma +1)}, \\ \overline{\mathcal{A}}_{k_{1}}(t,\alpha )= [\tilde{\delta }_{k} \tilde{\omega }_{k}^{\prime }(1-2\alpha )-(\hat{\gamma }_{k}^{\prime }+\tilde{m}_{k})(1-2 \alpha ) ]\frac{t^{\gamma }}{\varGamma (\gamma +1)}, \end{cases}\displaystyle \\ \end{aligned}$$

14 $$\begin{aligned}& \textstyle\begin{cases} \underline{\mathcal{R}}_{k_{1}}(t,\alpha )= (2\alpha -1) [ \hat{\gamma }_{k}+\hat{\gamma }_{k}^{\prime }-\tilde{m}_{k} ] \frac{t^{\gamma }}{\varGamma (\gamma +1)}, \\ \overline{\mathcal{R}}_{k_{1}}(t,\alpha )= (1-2\alpha ) [ \hat{\gamma }_{k}+\hat{\gamma }_{k}^{\prime }-\tilde{m}_{k} ] \frac{t^{\gamma }}{\varGamma (\gamma +1)}, \end{cases}\displaystyle \end{aligned}$$

15 $$\begin{aligned}& \textstyle\begin{cases} \underline{\mathcal{M}}_{k_{1}}(t,\alpha )= (2\alpha -1) [ \tilde{\xi }+\tilde{\eta }-\tilde{\nu } ] \frac{t^{\gamma }}{\varGamma (\gamma +1)}, \\ \overline{\mathcal{M}}_{k_{1}}(t,\alpha )= (1-2\alpha ) [ \tilde{\xi }+\tilde{\eta }-\tilde{\nu } ] \frac{t^{\gamma }}{\varGamma (\gamma +1)}. \end{cases}\displaystyle \end{aligned}$$

For the sake of simplicity, assume that

$$\begin{aligned}& \underline{C}_{1} = \tilde{n}_{k}- \tilde{m}_{k}(2\alpha -1)- \tilde{b}_{k}(2\alpha -1)^{2}-\kappa \tilde{b}_{k}(2\alpha -1)^{2}- \tilde{b}_{k}(2\alpha -1)^{2}, \\& \overline{C}_{1} = \tilde{n}_{k}- \tilde{m}_{k}(1-2\alpha )- \tilde{b}_{k}(1-2\alpha )^{2}-\kappa \tilde{b}_{k}(1-2\alpha )^{2}- \tilde{b}_{k}(1-2\alpha ), \\& \underline{C}_{2} = \tilde{b}_{k}(2\alpha -1)^{2}+\kappa \tilde{b}_{k}(2\alpha -1)^{2}+\tilde{b}_{k}(2\alpha -1)^{2}-(1- \tilde{\delta }_{k})\tilde{\omega }_{k}(2\alpha -1) \\& \hphantom{\underline{C}_{2} ={}}{} -\tilde{\delta }_{k}\tilde{\omega }_{k}^{\prime }(2 \alpha -1)-\tilde{m}_{k}(2 \alpha -1), \\& \overline{C}_{2} = \tilde{b}_{k}(1-2\alpha )^{2}+\kappa \tilde{b}_{k}(1-2 \alpha )^{2}+\tilde{b}_{k}(1-2\alpha )^{2}-(1- \tilde{\delta }_{k}) \tilde{\omega }_{k}(1-2\alpha ) \\& \hphantom{\overline{C}_{2} ={}}{} -\tilde{\delta }_{k}\tilde{\omega }_{k}^{\prime }(1-2 \alpha )-\tilde{m}_{k}(1-2 \alpha ), \\& \underline{C}_{3} = (1-\tilde{\delta }_{k})\tilde{ \omega }_{k}(2 \alpha -1)-(\hat{\gamma }_{p}+ \tilde{m}_{k}) (2\alpha -1), \\& \overline{C}_{3} = (1-\tilde{\delta }_{k})\tilde{ \omega }_{k}(1-2 \alpha )-(\hat{\gamma }_{p}+ \tilde{m}_{k}) (1-2\alpha ), \\& \underline{C}_{4} = \tilde{\delta }_{k}\tilde{ \omega }_{k}^{\prime }(2 \alpha -1)-\bigl(\hat{\gamma }_{k}^{\prime }+\tilde{m}_{k}\bigr) (2\alpha -1), \\& \overline{C}_{4} = \tilde{\delta }_{k}\tilde{ \omega }_{k}^{\prime }(1-2 \alpha )-\bigl(\hat{\gamma }_{k}^{\prime }+\tilde{m}_{k}\bigr) (1-2\alpha ), \\& \underline{C}_{5} = (2\alpha -1) \bigl[\hat{\gamma }_{k}+ \hat{\gamma }_{k}^{\prime }- \tilde{m}_{k} \bigr], \\& \overline{C}_{5} = (1-2\alpha ) \bigl[\hat{\gamma }_{k}+ \hat{\gamma }_{k}^{\prime }- \tilde{m}_{k} \bigr], \\& \underline{C}_{6} = (2\alpha -1) [\tilde{\xi }+\tilde{\eta }- \tilde{\nu } ], \\& \overline{C}_{6} = (1-2\alpha ) [\tilde{\xi }+\tilde{\eta }- \tilde{\nu } ]. \end{aligned}$$

Now the third term of the series is

16 $$ \textstyle\begin{cases} \underline{\mathcal{Y}}_{k_{2}}(t,\alpha )= \tilde{n}_{k} \frac{t^{\gamma }}{\varGamma (\gamma +1)}-\tilde{m}_{k}\underline{C}_{1} \frac{t^{2\gamma }}{\varGamma (2\gamma +1)} [\tilde{b}_{k}(2 \alpha -1)\underline{C}_{3}+\underline{C}_{1} ] \frac{t^{2\gamma }}{\varGamma (2\gamma +1)} \\ \hphantom{\underline{\mathcal{Y}}_{k_{2}}(t,\alpha )={}}{} - [\tilde{b}_{k}(2\alpha -1)(\underline{C}_{6}+\underline{C}_{1}) ]\frac{t^{2\gamma }}{\varGamma (2\gamma +1)}, \\ \overline{\mathcal{Y}}_{k_{2}}(t,\alpha )= \tilde{n}_{k} \frac{t^{\gamma }}{\varGamma (\gamma +1)}-\tilde{m}_{k}\overline{C}_{1} \frac{t^{2\gamma }}{\varGamma (2\gamma +1)} [\tilde{b}_{k}(1-2 \alpha )\overline{C}_{3}+\overline{C}_{1} ] \frac{t^{2\gamma }}{\varGamma (2\gamma +1)} \\ \hphantom{\overline{\mathcal{Y}}_{k_{2}}(t,\alpha )={}}{} - [\tilde{b}_{k}(1-2\alpha )(\overline{C}_{6}+\overline{C}_{1}) ]\frac{t^{2\gamma }}{\varGamma (2\gamma +1)}, \\ \underline{\mathcal{V}}_{k_{2}}(t,\alpha )= [\tilde{b}_{k}(2 \alpha -1)(\underline{C}_{3}+\underline{C}_{1}) ] \frac{t^{2\gamma }}{\varGamma (2\gamma +1)}+ [\tilde{b}_{k} \kappa (2\alpha -1)(\underline{C}_{4}+\underline{C}_{1}) ] \frac{t^{2\gamma }}{\varGamma (2\gamma +1)} \\ \hphantom{\underline{\mathcal{V}}_{k_{2}}(t,\alpha )={}}{} -\underline{C}_{2} [(1-\tilde{\delta }_{k})-\tilde{\delta }_{k} \tilde{\omega }_{k}^{\prime }-\tilde{m}_{k} ] \frac{t^{2\gamma }}{\varGamma (2\gamma +1)}, \\ \overline{\mathcal{V}}_{k_{2}}(t,\alpha )= [\tilde{b}_{k}(1-2 \alpha )(\overline{C}_{3}+\overline{C}_{1}) ] \frac{t^{2\gamma }}{\varGamma (2\gamma +1)}+ [\tilde{b}_{k} \kappa (1-2\alpha )(\overline{C}_{4}+\overline{C}_{1}) ] \frac{t^{2\gamma }}{\varGamma (2\gamma +1)} \\ \hphantom{\overline{\mathcal{V}}_{k_{2}}(t,\alpha )={}}{} -\overline{C}_{2} [(1-\tilde{\delta }_{k})-\tilde{\delta }_{k} \tilde{\omega }_{k}^{\prime }-\tilde{m}_{k} ] \frac{t^{2\gamma }}{\varGamma (2\gamma +1)}, \\ \underline{\mathcal{I}}_{k_{2}}(t,\alpha )= (1-\tilde{\delta }_{k}) \tilde{\omega }_{k}\underline{C}_{2} \frac{t^{2\gamma }}{\varGamma (2\gamma +1)}-(\hat{\gamma }_{k}+ \tilde{m}_{k})\underline{C}_{3} \frac{t^{2\gamma }}{\varGamma (2\gamma +1)}, \\ \overline{\mathcal{I}}_{k_{2}}(t,\alpha )= (1-\tilde{\delta }_{k}) \tilde{\omega }_{k}\overline{C}_{2} \frac{t^{2\gamma }}{\varGamma (2\gamma +1)}-(\hat{\gamma }_{k}+ \tilde{m}_{k})\overline{C}_{3} \frac{t^{2\gamma }}{\varGamma (2\gamma +1)}, \\ \underline{\mathcal{A}}_{k_{2}}(t,\alpha )= \tilde{\delta }_{k} \tilde{\omega }_{k}^{\prime }\underline{C}_{2} \frac{t^{2\gamma }}{\varGamma (2\gamma +1)}-(\hat{\gamma }_{k}^{\prime }+ \tilde{m}_{k})\underline{C}_{4} \frac{t^{2\gamma }}{\varGamma (2\gamma +1)}, \\ \overline{\mathcal{A}}_{k_{2}}(t,\alpha )= \tilde{\delta }_{k} \tilde{\omega }_{k}^{\prime }\overline{C}_{2} \frac{t^{2\gamma }}{\varGamma (2\gamma +1)}-(\hat{\gamma }_{k}^{\prime }+ \tilde{m}_{k})\overline{C}_{4} \frac{t^{2\gamma }}{\varGamma (2\gamma +1)}, \\ \underline{\mathcal{R}}_{k_{2}}(t,\alpha )= (\hat{\gamma }_{k} \underline{C}_{3}-\hat{\gamma }_{k}^{\prime }\underline{C}_{4}-\tilde{m}_{k} \underline{C}_{5})\frac{t^{2\gamma }}{\varGamma (2\gamma +1)}, \\ \overline{\mathcal{R}}_{k_{2}}(t,\alpha )= (\hat{\gamma }_{k} \overline{C}_{3}-\hat{\gamma }_{k}^{\prime }\overline{C}_{4}-\tilde{m}_{k} \overline{C}_{5})\frac{t^{2\gamma }}{\varGamma (2\gamma +1)}, \\ \underline{\mathcal{M}}_{k_{2}}(t,\alpha )= (\tilde{\xi } \underline{C}_{3}-\tilde{\eta }\underline{C}_{4}-\tilde{\upsilon } \underline{C}_{6})\frac{t^{2\gamma }}{\varGamma (2\gamma +1)}, \\ \overline{\mathcal{M}}_{k_{2}}(t,\alpha )= (\tilde{\xi }\overline{C}_{3}- \tilde{\eta }\overline{C}_{4}-\tilde{\upsilon }\overline{C}_{6}) \frac{t^{2\gamma }}{\varGamma (2\gamma +1)}. \end{cases} $$

**Figure 1:**
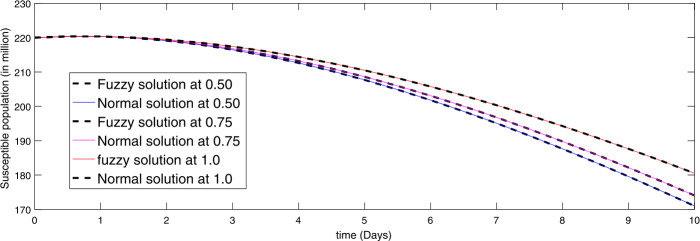
Comparison of approximate fuzzy and normal solution for susceptible compartment for three terms at the given uncertainty values $\alpha \in [0, 1]$ against various fractional order

**Figure 2:**
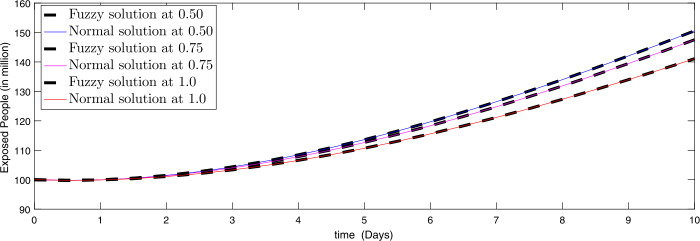
Comparison of approximate fuzzy and normal solution for exposed compartment for three terms at the given uncertainty values $\alpha \in [0, 1]$ against various fractional order

**Figure 3:**
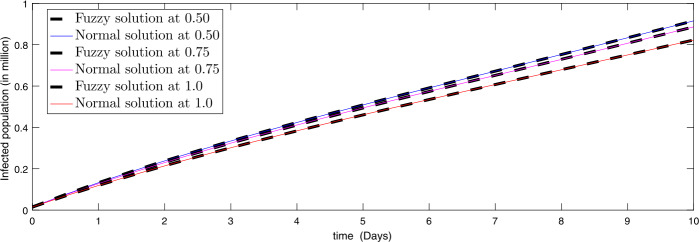
Comparison of approximate fuzzy and normal solution for infected compartment for three terms at the given uncertainty values $\alpha \in [0, 1]$ against various fractional order

**Figure 4:**
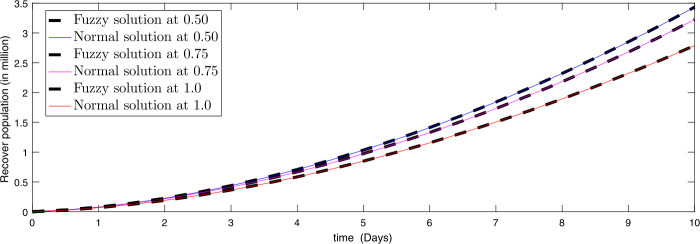
Comparison of approximate fuzzy and normal solution for recovered compartment for three terms at the given uncertainty values $\alpha \in [0, 1]$ against various fractional order

**Figure 5:**
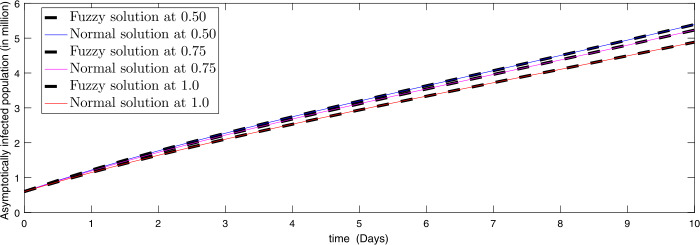
Comparison of approximate fuzzy and normal solution for asymptotically infected compartment for three terms at the given uncertainty values $\alpha \in [0, 1]$ against various fractional order

**Figure 6:**
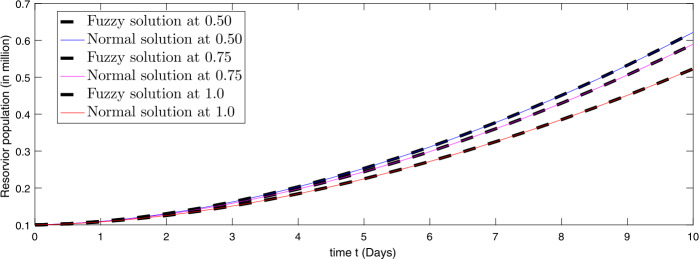
Comparison of approximate fuzzy and normal solution for reservoir compartment for three terms at the given uncertainty values $\alpha \in [0, 1]$ against various fractional order

In Figs. 1–6 we presented comparisons of approximate fuzzy and approximate normal solutions for the considered model at the given uncertainty against various fractional order. We see that as the susceptible class value is decreasing, the exposed papulation increases and hence infection spreads with different rate due to various fractional order. Similarly, the death cases are increasing so the recovered class also grows and the asymptotically infected class also increases, and hence the population of virus in the reservoir is growing. From the figures we observe that fuzzyness along with fractional calculus provides global dynamics to such a kind of nonlinear problems where uncertainty lies in the data.

### Remark 1

Regarding the provided results, it is clear that the lower bound is an increasing set-valued function and an upper bound is a decreasing one, which proves that the solutions are fuzzy numbers. Also, it is worthy to mention that for general cases, similar results can be obtained under fuzzy differentiability.

### Remark 2

Considering the fact that stochastic and random parameters are more complex to address, the uncertainty can lead to an increase in the computation cost, so employing fuzzy concepts for modeling such real-world systems can be the most suitable choice.

## Conclusions

In this paper, we have demonstrated the existence and uniqueness of the solution to the fuzzy fractional order model of COVID-19 infection using the Banach fixed point theorem. We also established a proper procedure for the fuzzy Laplace transform coupled with Adomian decomposition method to obtain an approximate solution for the proposed model. We have presented comparisons between fuzzy and normal results up to three terms to depict the efficiency of this approach. We observed that fuzzyness coupled with fractional calculus approach excellently produced global dynamics of those problems where uncertainty lies in the data.

## References

[1] Jasper, F.W.C., Kin, H.K., Zheng, Z.: Genomic characterization of the 2019 novel human-pathogenic coronavirus isolated from patients with acute respiratory disease in Wuhan, Hubei, China. Emerg. Microbes Infect., 1–50 (2020) 10.1080/22221751.2020.1719902 PMC7067204 31987001

[2] Lu H., Stratton C.W., Tang Y.W. (2020). Outbreak of pneumonia of unknown etiology in Wuhan, China: the mystery and the miracle. J. Med. Virol..

[3] Ji, W., Wang, W., Zhao, X., Zai, J., Li, X.: Homologous recombination within the spike glycoprotein of the newly identified coronavirus may boost cross-species transmission from snake to human. J. Med Virol. **268** (2020). 10.1002/jmv.25682

[4] World Health Organization: coronavirus disease 2019 (COVID-19) situation report-62, 270. https://www.who.int/docs/default-source/coronaviruse/situation-reports/20200322-sitrep-62-covid-27119

[5] Chen Y., Guo D. (2016). Molecular mechanisms of coronavirus RNA capping and methylation. Virol. Sin..

[6] Wang L.F. (2006). Review of bats and SARS. Emerg. Infect. Dis..

[7] Ge X.Y. (2013). Isolation and characterization of a bat SARS-like coronavirus that uses the ACE2 receptor. Nature.

[8] Lu R., Zhao X., Li J., Niu P., Yang B., Wu H., Tan W. (2020). Genomic characterisation and epidemiology of 2019 novel coronavirus: implications for virus origins and receptor binding. Lancet.

[9] Zhou P., Yang X.L., Wang X.G., Hu B., Zhang L., Zhang W., Shi Z.L., Si H.R., Zhu Y., Li B., Huang C.L. (2020). A pneumonia outbreak associated with a new coronavirus of probable bat origin. Nature.

[10] Tian X., Li C., Huang A., Xia S., Lu S., Shi Z., Lu L., Jiang S., Yang Z., Wu Y., Ying T. (2020). Potent binding of 2019 novel coronavirus spike protein by a SARS coronavirus-specific human monoclonal antibody. Emerg. Microbes Infect..

[11] Ahmed S.F., Quadeer A.A., McKay M.R. (2020). Preliminary identification of potential vaccine targets for 2019-nCoV based on SARS-CoV immunological studies. Viruses.

[12] Chaudhury S., Berrondo M., Weitzner B.D., Muthu P., Bergman H., Gray J.J. (2011). Benchmarking and analysis of protein docking performance in Rosetta. PLoS ONE.

[13] Khan M.A., Atangana A. (2020). Modeling the dynamics of novel coronavirus (2019-nCov) with fractional derivative. Alex. Eng. J..

[14] Agarwal R.P., Lakshmikantham V., Nieto J.J. (2010). On the concept of solution for fractional differential equations with uncertainty. Nonlinear Anal..

[15] Asjad M.I., Aleem M., Ahmadian A., Salahshour S., Ferrara M. (2020). New trends of fractional modeling and heat and mass transfer investigation of (SWCNTs and MWCNTs)-CMC based nanofluids flow over inclined plate with generalized boundary conditions. Chin. J. Phys..

[16] Aleem M., Asjad M.I., Chowdhury M.S.R., Hussanan A. (2019). Analysis of mathematical model of fractional viscous fluid through a vertical rectangular channel. Chin. J. Phys..

[17] Asjad M.I., Aleem M., Ahmadian A., Salimi M., Ferrara M. (2020). Heat transfer analysis of channel flow of MHD Jeffrey fluid subject to generalized boundary conditions. Eur. Phys. J. Plus.

[18] Imran M.A., Aleem M., Riaz M.B., Ali R., Khan I. (2019). A comprehensive report on convective flow of fractional (ABC) and (CF) MHD viscous fluid subject to generalized boundary conditions. Chaos Solitons Fractals.

[19] Kaleva O. (1987). Fuzzy differential equations. Fuzzy Sets Syst..

[20] Lupulescu V. (2015). Fractional calculus for interval-valued functions. Fuzzy Sets Syst..

[21] Arshad S., Luplescu V. (2011). Fractional differential equation with fuzzy initial condition. Electron. J. Differ. Equ..

[22] Benchohra M., Cabada A., Seba D. (2009). An existence result for nonlinear fractional differential equations on Banach spaces. Bound. Value Probl..

[23] Belmekki M., Nieto J.J., Lopez R.R. (2009). Existence of periodic solution for a nonlinear fractional differential equation. Bound. Value Probl..

[24] Park J.Y., Kwan Y.C., Jeong J.V. (1999). Existence and uniqueness theorem for a solution of fuzzy Volterra integral equations. Fuzzy Sets Syst..

[25] Ali N., Khan R.A. (2016). Existence of positive solution to a class of fractional differential equations with three point boundary conditions. Math. Sci. Lett..

[26] Khan R.A., Shah K. (2015). Existence and uniqueness of solutions to fractional order multi-point boundary value problems. Commun. Appl. Anal..

[27] Lakshmikantham V., Leela S. (2009). Naguma-type uniqueness result for fractional differential equations. Nonlinear Anal..

[28] Miller K.S., Ross B. (1993). An Introduction to the Fractional Calculus and Fractional Differential Equations.

[29] Lakshmikantham V., Vatsala A.S. (2008). Basic theory of fractional differential equations. Nonlinear Anal..

[30] Perfilieva I. (2006). Fuzzy transforms: theory and applications. Fuzzy Sets Syst..

[31] Salahshour S., Allahviranloo T., Abbasbandy S. (2012). Solving fuzzy fractional differential equations by fuzzy Laplace transform. Commun. Nonlinear Sci. Numer. Simul..

[32] Allahviranloo T., Salahshour S., Abbasbandy S. (2012). Explicit solutions of fractional differential equations with uncertainty. Soft Comput..

[33] Allahviranloo T., Ahmadi M.B. (2010). Fuzzy Laplace transform. Soft Comput..

[34] Zhu Y. (2010). Stability analysis of fuzzy linear differential equations. Fuzzy Optim. Decis. Mak..

[35] Zimmermann H.J. (1991). Fuzzy Set Theory and Its Applications.

[36] Zadeh L.A. (1965). Fuzzy sets. Inf. Control.

